# The potential role of n-3 fatty acids and their lipid mediators on asthmatic airway inflammation

**DOI:** 10.3389/fimmu.2024.1488570

**Published:** 2024-12-10

**Authors:** Yuan Tian, JingMeng Sun, DongMei Jiao, WeiYu Zhang

**Affiliations:** ^1^ School of Pharmacy, Changchun University of Traditional Chinese Medicine, Changchun, China; ^2^ Department of Pharmacy, First Hospital of Jilin University, Changchun, China; ^3^ Analytical Preparation Process Department, Shouyao Holdings (Beijing) Co., Ltd, Beijing, China

**Keywords:** asthma, inflammation, n-3 fatty acid, maresin, protectin, resolvin

## Abstract

Asthma, is a common, significant and diverse condition marked by persistent airway inflammation, with a major impact on human health worldwide. The predisposing factors for asthma are complex and widespread. The beneficial effects of omega-3 (n-3) polyunsaturated fatty acids (PUFAs) in asthma have increasingly attracted attention recently. In asthma therapy, n-3 PUFAs may reduce asthma risk by controlling on levels of inflammatory cytokines and regulating recruitment of inflammatory cells in asthma. The specialized pro-resolving mediators (SPMs) derived from n-3 PUFAs, including the E- and D-series resolvins, protectins, and maresins, were discovered in inflammatory exudates and their biosynthesis by lipoxygenase mediated pathways elucidated., SPMs alleviated T-helper (Th)1/Th17 and type 2 cytokine immune imbalance, and regulated macrophage polarization and recruitment of inflammatory cells in asthma via specific receptors such as formyl peptide receptor 2 (ALX/FPR2) and G protein-coupled receptor 32. In conclusion, the further study of n-3 PUFAs and their derived SPMs may lead to novel anti-inflammatory asthma treatments.

## 1 Introduction

Asthma, a common, non-communicable condition, with substantial morbidity, impacted 262 million individuals worldwide and resulted in 455,000 deaths, according to recent analyses (1, 2). The diversity and universality of pathogenic factors account for its widespread prevalence. Genetic risk factors, including family history and gender; lifestyle factors such as diet, exercise, stress, obesity and environmental factors, particularly inhalant allergens (dust mites, pollen), air pollution, smoke and occupational exposures (3), all affect the prevalence and mortality of asthma globally (4). The pathogenesis of asthma is extremely complex, involving multiple inflammatory mechanisms including Type 2 inflammation, T-helper (Th)1/Th17 immune imbalance, increased inflammatory cytokines, inflammatory cell recruitment and ultimately pathologic changes in the airways (5).

Omega-3 (n-3) polyunsaturated fatty acids (PUFAs) comprise a group of polyunsaturated fats, represented by Docosahexaenoic acid (DHA), eicosapentaenoic acid (EPA), essential nutrients, found in many foods. Published research suggests that n-3 PUFAs exhibit immunologic activity, affecting a variety of physiologic and pathologic processes, including cognitive function (6), vascular and myocardial function (7), inflammation (8), atopic disease (9), and cardiovascular diseases (10). In recent years, n-3 PUFAs-derived lipid mediators called specialized pro-resolving mediators (SPMs) were discovered and found to be biosynthesized by lipoxygenase mediated pathways, with the reports on their pro-resolving effects and anti-inflammatory activity. SPMs were able to regulate various inflammatory mechanisms in asthma and were the potential active mediators of the anti-asthma effects of n-3 PUFAs (11). This review aims to assess the established benefits of n-3 PUFAs in asthma, focusing on the n-3 PUFA-derived specialized pro-resolving lipid mediators and their anti-inflammatory properties.

## 2 Asthma phenotyping

Asthma is a diverse condition characterized by fluctuating respiratory symptoms, particularly wheeze, cough and breathlessness, which vary in intensity and frequency over time, associated with reversible expiratory airflow limitation, which may persist and become irreversible (12). Among the primary pathologic traits of asthma are airway hyper-responsiveness (AHR), airway remodeling, disrupted mucosal immunity, and persistent airway inflammation (13, 14).

Asthma has been classified into different phenotypes (15): according to age (childhood (16), adolescent (17), adult (18), and elderly asthma (19)); severity (severe and non-severe asthma (20)); inducing factors (allergic, non-allergic and occupational asthma (12), obesity asthma (21), etc.); biomarkers (eosinophilic, neutrophilic asthma, etc. (22)). Cluster analysis studies have defined the main phenotypes of asthma including early-onset allergic asthma, early-onset allergic moderate-to-severe remodeled asthma, late-onset nonallergic eosinophilic asthma, and late-onset nonallergic noneosinophilic asthma etc. (23). Endotypes, subtypes of disease defined functionally and pathologically by a molecular mechanism or by treatment, more succinctly classify asthma as type 2 (T2) and non-T2 types (24).

## 3 T2 asthma

**Figure 1:**
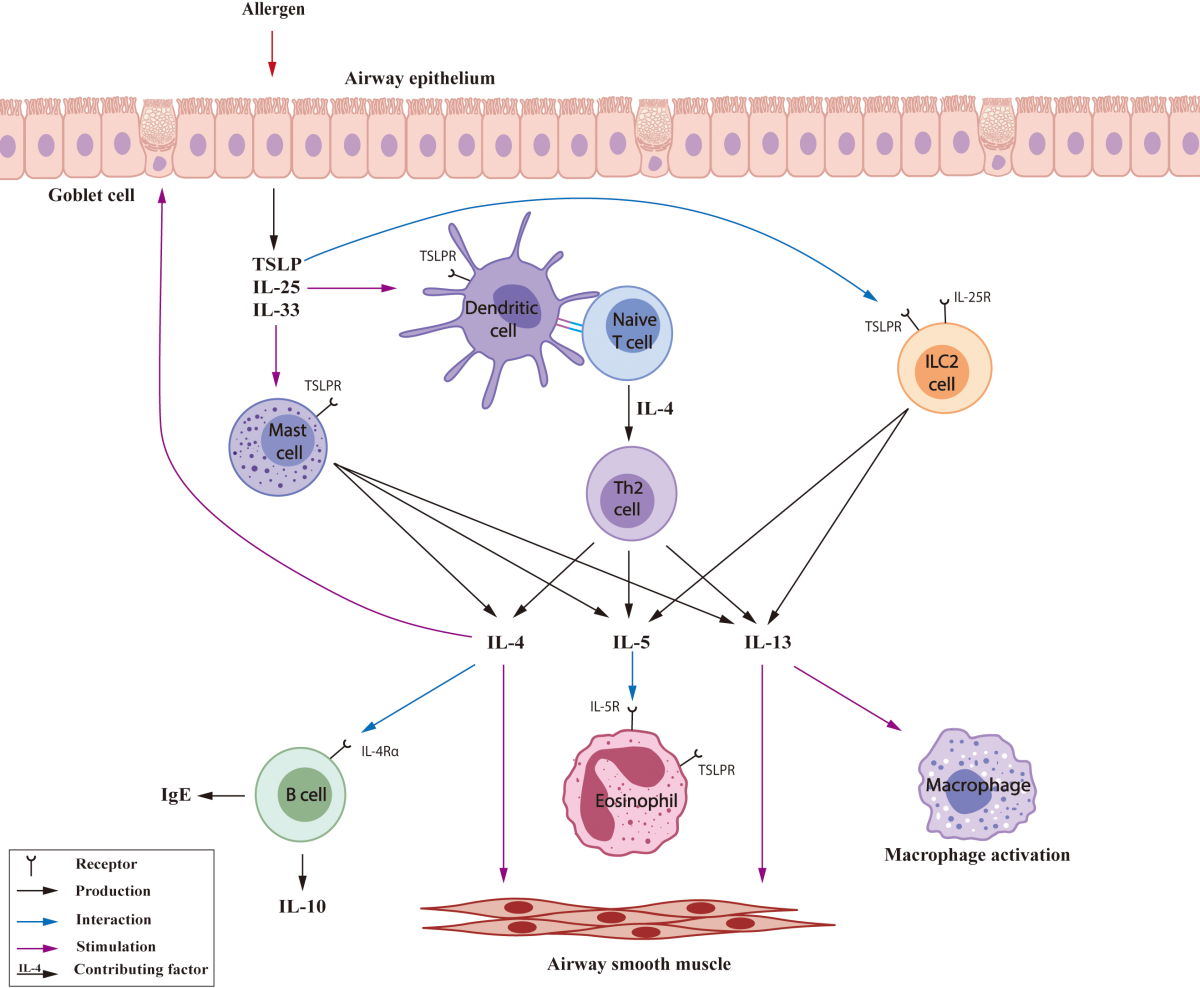
T2 inflammatory mechanisms in asthma. TSLP: thymic stromal lymphopoietin.

Type 2 immune processes represent a classic mechanism of allergy and an essential feature of asthma (**Figure 1**). Type 2 inflammation plays a major role in eosinophilic and allergic asthma, and has been observed in 50% - 70% asthma patients (25). Inhaled allergens stimulate airway epithelial cells to release alarmins (26), which may interact with dendritic cells (DCs) and induce differentiation of naive T cells into Th2 cells (27). In addition, Th2 cells and type 2 innate lymphoid cells (ILC2s) produce a variety of type 2 cytokines, especially interleukins including interleukin (IL)-4, IL-5 and IL-13 (28, 29). IL-4 promotes the differentiation of Th2 cells, B cell switching and IgE production, goblet cell hyperplasia and mucus production, epithelial barrier disruption and tissue remodeling, airway smooth contraction and AHR (30, 31). Although the major effects of IL-13 are very similar to those of IL-4, some independent pathways of eosinophilia (32) and M2 macrophage polarization (33) have been reported for IL-13. IL-5 has a pivotal role in facilitating the maturation and recruitment of eosinophils (34); it is also released by mast cells and ILC2s, particularly after interaction with thymic stromal lymphopoietin (TSLP) (33, 35). Mixed granulocytic asthma, with elevation of sputum (and airway) neutrophils and eosinophils is a rarer phenotype, but it tends to feature Type 2 inflammation with the anticipated responses (22, 36).

## 4 Non-T2 asthma

**Figure 2:**
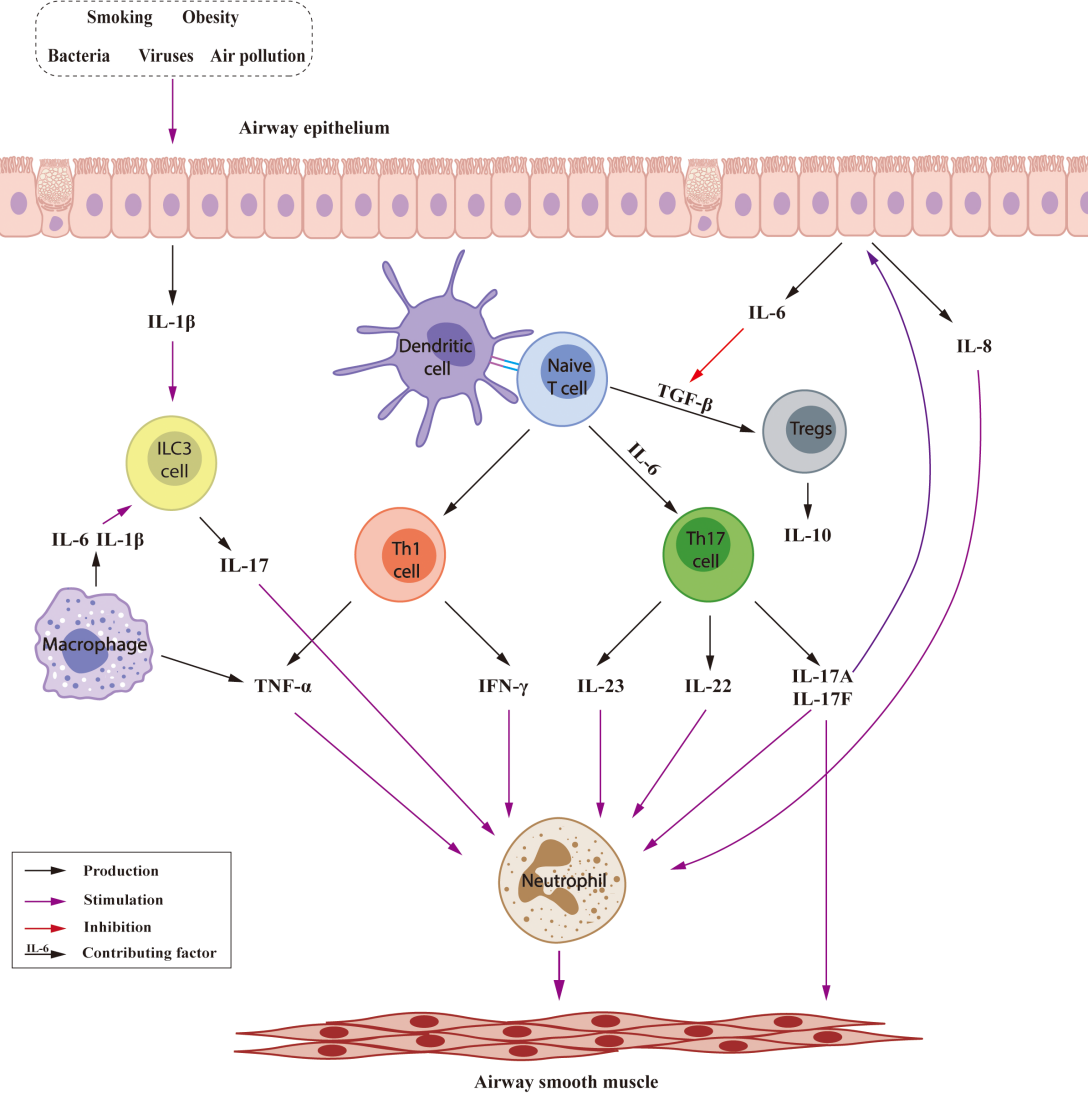
Inflammatory mechanisms involved in Non-T2 asthma.

Non-T2 asthma is characterized by neutrophilic and paucigranulocytic inflammation, and may be triggered by factors including smoking, obesity, bacteria, viruses, and air pollution (**Figure 2**) (37). In non-T2 asthma, naive T cells differentiate into Th1, Th17 cells. Th1 cells produce tumor necrosis factor-α (TNF-α) and interferon-gamma (IFN-γ) while Th17 cells produce a variety of cytokines (38, 39); together contributing to recruitment and activation of neutrophils leading to AHR and airway remodeling (40, 41). IL-17 stimulated airway epithelial cells release IL-6, which promotes differentiation of naive T cells into Th17 cells (42) and inhibition of transforming growth factor-β (TGF-β)-induced production of regulatory T cells (Tregs) (43). IL-8, is also produced by airway epithelial cells, increasing neutrophil numbers (44). In addition, innate lymphoid cells (ILC) 3 cells are another source of IL-17 (45), and macrophage-derived IL-6 and IL-1β could stimulate ILC3 to produce IL-17 (46, 47). Tregs, generated from naive T cells, suppress the Th2 response in asthma, inhibition TGF-β may exacerbate airway inflammation and remodeling by Treg downregulation (48). Tregs (49), B cells (50) and CD8+ T cells (51) produce IL-10, which decrease tissue mast cell and eosinophil counts and may prevent neutrophilic asthma.

Paucigranulocytic asthma may account for up to 40% of patients with asthma (52) and though it was usually well controlled on treatment, or intermittent in the Severe Asthma Research Program cohort (53), it has been relatively little studied. It has been suggested that the number of granulocytes may reflect depletion of eosinophils by steroid therapy. By contrast with the immune imbalance in neutrophilic asthma,paucigranulocytic asthma may be more strongly associated with neural regulation as suggested by high levels of nerve growth factor (NGF) (54) and sphingolipid synthesis inhibition (55) induced AHR, and bronchoconstrictor signaling (56) are also involved in the pathogenesis of paucigranulocytic asthma.

## 5 PUFAs in asthma

**Figure 3:**
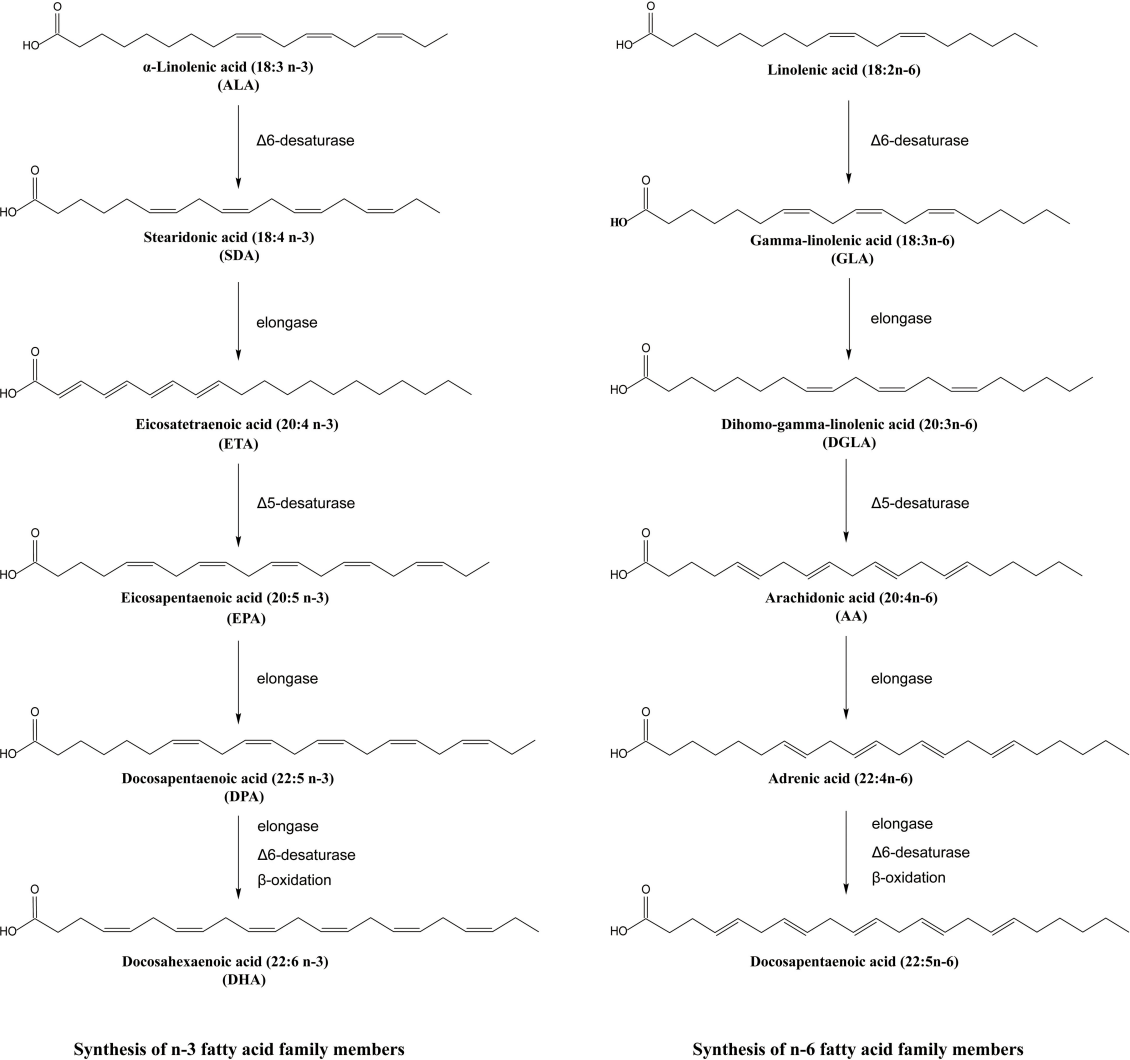
Synthesis of n-3 and n-6 fatty acid family members.

PUFAs are defined as fatty acids characterized by the presence of multiple double bonds, with a terminal methyl carbon at one end and the iconic hydroxyl group at the other (57). They are sometimes called essential fatty acids as they cannot be synthesized by humans and must be obtained through the diet. PUFAs are classified as omega-3 or n-3 PUFAs when their first double bond is situated between the third and fourth carbon atoms (58) and omega-6 PUFAs when the carbon-carbon double bond is at the n-6 position. A series of enzymatic reactions catalyzed the synthesis of n-3 PUFAs from the precursor alpha-linoleic acid (ALA), including EPA, DHA, and docosapentaenoic acid (DPA) and the biosynthesis of n-6 PUFAs including gamma-γ-linolenic acid (GLA), dihomo-gamma-linolenic acid (DGLA), and arachidonic acid (AA) (59, 60) as shown in **Figure 3**. The Δ5 desaturase and the Δ6 desaturase enzymes insert double bonds at the fifth and sixth carbon atoms, and the chain is shorted by β-oxidation (61). The shared desaturase and elongase enzymes lead to competition between n-3 and n-6 PUFAs, the n-6/n-3 ratio in organisms sometimes depends on the ingested ratio of substrates for n-6 and n-3 PUFAs (62).The importance of the n-6/n-3 ratio has been highlighted in cardiovascular disease (63), cancer (64), asthma (65) and other diseases. Because of the complicated combined actions of n-3 and n-6 PUFAs, beneficial effects of mixed fatty acids at an n-6/n-3 ratio of 5:1 were reported in asthma but at a ratio of 10:1, the effects became negative (66), suggesting meaningful roles for both n-3 and n-6 PUFAs in asthma.

**Figure 4:**
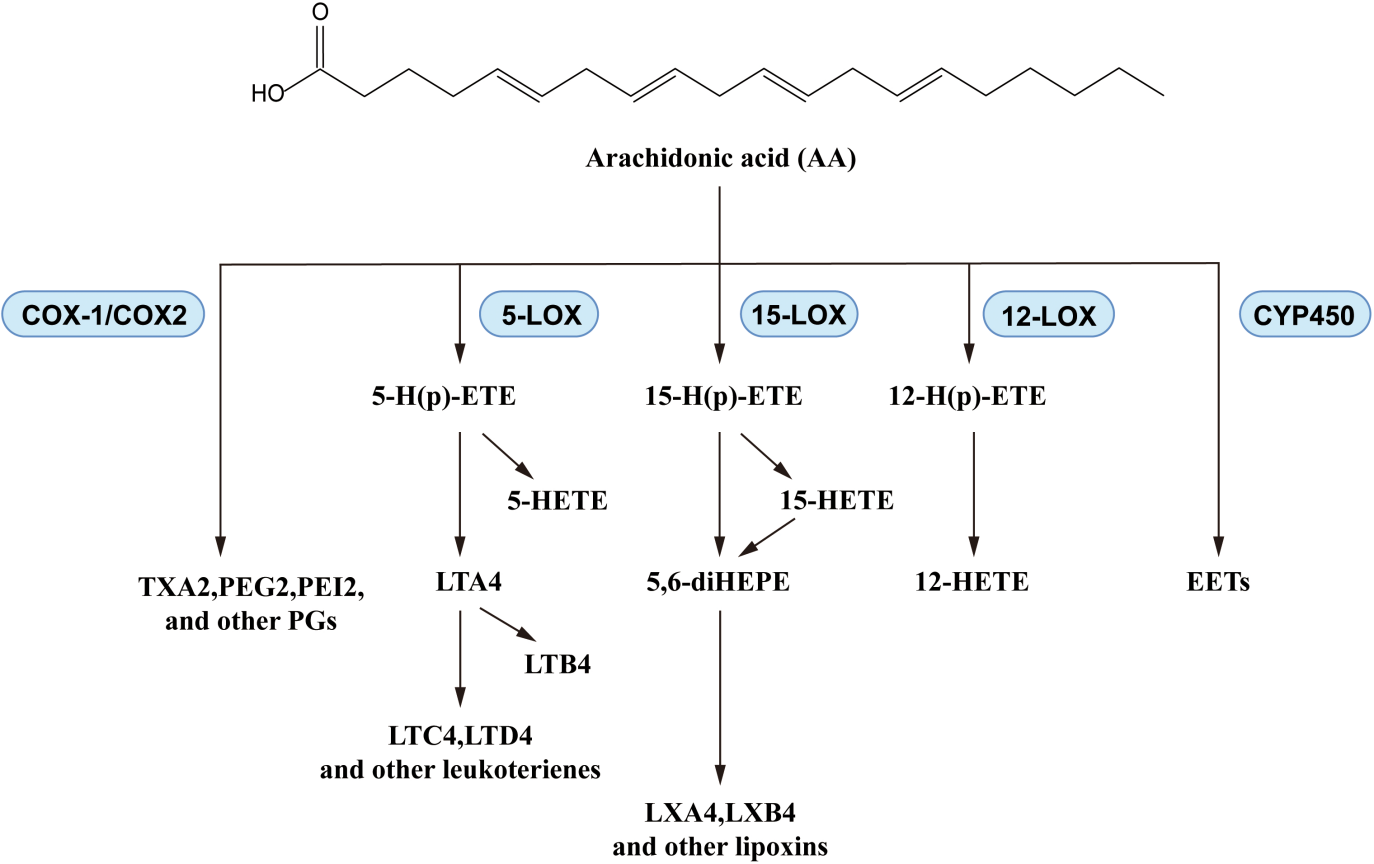
Biosynthesis of AA-derived lipid mediators.

N-6 PUFAs, particularly AA, have demonstrated complex effects in asthma. In a large cross-sectional study, asthma risk was significantly negatively corelated with omega-6 fatty acid intake (67), as in the report from Lee-Sarwar et al. (9). However, asthma exacerbations influenced the levels of n-6 PUFAs *in vivo*: the plasma AA levels showed a positive correlation with childhood asthma attacks (68). Similar trends were also observed in lung cells of asthmatic mice (69), and in plasma levels of the AA-derived eicosanoids, prostaglandin E2 (PGE2) and thromboxane B2 (TXB2), in asthma patients (70). N-6 PUFAs generate mediators that play important roles in asthma development (71, 72), while AA produces leukotrienes, prostaglandins, and thromboxanes via a series of enzymatic reactions catalyzed by cyclooxygenase and lipoxygenase (**Figure 4**). There are some reports about the pro-inflammatory activities of eicosanoids: leukotrienes increased vascular permeability and smooth-muscle contraction (73), prostaglandins induced allergen sensitization and Th2 immune response (74), and thromboxanes promoted bronchoconstriction and AHR (75). Taking into consideration the positive regulatory effect of n-6 PUFAs in asthma, further studies are needed to clarify the complex mechanisms of n-6 PUFAs effects in asthma. In fact, existing studies of PUFAs in asthma are more focused on the n-3 PUFAs: many clinical trials and animal experiments have elucidated their effects.

## 6 Effects of n-3 PUFAs in asthma and lung inflammation

As critical nutrients in diets, the sources of n-3 PUFAs are multifarious. The main sources of EPA and DPA are fish and seafood, while ALA is found in leafy vegetables and nuts (57). DHA has played a beneficial role in cardiovascular disease, the brain and visual function and inflammation (76). EPA showed helpful influences on brain function, oxidative stress, inflammation, hyperlipidemia and neurodegenerative diseases (77). Fish and lean red meat are sources of DPA, and the effects of DPA such as anti-inflammatory actions, antiplatelet aggregation, and improvement of plasma lipid have been reported (78). Because of the high β-oxidation rate of ALA (79), the few sources and low conversion of SDA to DHA (80), these two kinds of n- 3 PUFAs are rarely used in clinical anti-inflammatory treatment. In this review, we mainly discuss EPA, DHA, and DPA that are easily obtained in the daily diet and frequently supplemented in asthma therapy. Positive outcomes associated with n-3 PUFAs have been documented in the context of preventive measures (81, 82) and disease control (83) of asthma. According to a related study (84), n-3 intake decreased asthma risk in a dose-dependent manner (< 59.0 mg/kg/day). Various types of n-3 PUFA supplements have been implemented; including the delivery of combinations of various PUFAs, fish oil and diets rich in PUFAs. This article will examine the impact of various forms of n-3 PUFAs, rather than n-6 PUFAs, on asthma prevalence, lung inflammation, asthma challenge testing, and clinical asthma, as reported in recent clinical trials [**Table 1** (85–88), **Table 2** (89–97)] and in animal/cellular asthma models [**Table 3** (69, 98–103)].

**Table 1:** Effects of n-3 PUFAs on asthma in clinical trials.

Form of n-3 PUFAs	Intervention	Participants	Outcomes	Reference
EPA and DHA	High dose (3.7 g EPA + 2.5 g DHA/d) x 21 days, low dose (1.8 g EPA + 1.3 g DHA/d) and placebo x 21 days	8 male adults with asthma and HIB and 8 healthy male adult controls	Peak fall in FEV_1_ reduced by 34% and 30% (both p + 0.001): baseline fraction of exhaled NO was reduced by 24% and 31% (p = or < 0.02)	(85)
EPA and DHA	180 mg EPA + 120 mg DHA/d x 3 months	39 asthma patients (aged 4 - 14 y)	Two-point improvement in symptom score in 28 patients and in PEF and lower IL-17A and TNF-α levels (p < 0.05)	(86)
PUFA-enriched fat blend	450 mg EPA + 180 mg DHA + 60 mg gamma linoleic acid + 60 mg SDA/d	13 female and 10 male adults with asthma (aged 22 - 29 y)	eNO was significantly lower (p = 0.022) with lower levels of serum eosinophils (10.1 8 ± 0.1.84 vs. 5.79 ± 80.69%), eosinophilic cationic protein (20.5 8 ± 9.93 vs. -1.68 ± 4.36 ng/mL) and cysteinyl leukotriene release (2,889 ± 872 vs. 1,120 ± 173 ng/mL) (p < 0.05 each) in the n-3 PUFA group	(87)
EPA and DHA	(55% EPA+37% DHA) 2.4 g/day and placebo from 24 weeks’ gestation until 1week postpartum	Pregnant women and their offspring between birth and 3 to 5 years of age	The cumulative risk of persistent wheeze or asthma were decreased from birth to 3 - 5 years (16.9% vs 23.7%, relative risk (RR) = 0.69, 95%CI 0.49 to 0.97, p = 0.035) and birth to 5 years (17.5% vs 24.6%, RR = 0.68, 95%CI 0.49 to 0.95, p = 0.024), but no difference in the risk of asthma exacerbations	(88)

HIB, hyperpnoea-induced bronchoconstriction; FEV_1_, forced expiratory volume in 1 second; eNO, exhaled nitric oxide; y, years; PEF, peak expiratory flow; ELFE, Etude Longitudinale Francais depuis L’Enfance; LCPUFA, long-chain PUFA; RR, relative risk.

**Table 2:** Effects of n-3 PUFA-enriched fish oil diets on asthma in clinical trials.

Form of n-3 PUFAs	Intervention	Participants	Outcomes	Reference
EPA, DHA and DPA in diet	Median dietary intakes: 0.24 g/1900 kcal and 0.27 g/1900 kcal at 8 and 16 y	1992 children born between 1994 and 1996 Swedish population cohort	High plasma n-3 PUFAs at 8 y was inversely associated with prevalent asthma at 24 y suggesting protection from lower dietary intake in childhood	(89)
Diet with enriched DHA or EPA	9.0 to 20.3 mg/100 kcal for DHA and 2.1 to 3.3 mg/100 kcal for EPA	8389 formula-fed infants from ELFE cohort at 2 mouths 36% had DHA and ARA supplementation and 11% had DHA, ARA and EPA supplementation	High DHA, ARA and EPA content supplementation was associated with a lower use of asthma medications from aged 2 months to 5.5 y	(90)
n-3 PUFAs in diet estimated by food questionnaire	1.6 - 2.6 g/d total n-3 PUFA	412 mother-child dyads (22% with active asthma in pregnancy)	Lower n-3 PUFA intake during pregnancy was significantly associated with risk of childhood asthma to age 4 y (p < 0.03), more apparent in female children	(91)
n-3 PUFA-rich fish oil	(55% EPA + 37% DHA) 2.4 g/day and placebo in 3^rd^ trimester, double-blind and randomized	695 pregnant women and their children followed up till age 6 y	n-3 PUFA supplementation in 3^rd^ trimester reduced risk of wheeze or asthma by 30% at 6 y with 73% reduction in non-atopic asthma	(92)
n-3 LCPUFA-rich fish oil	(800 mg DHA + 100 mg EPA)/d double-blind randomized multicenter recruited from 21 weeks of pregnancy onwards	706 Australian children with a family history of allergic disease followed up till 6y of age	No difference in the percentage of children with allergic disease (RR 1.04 p = 0.73)	(93)
n-3 PUFA-rich fish oil	Fish oil dose < 250 mg in 11, Fish oil dose < 250 and < 500 mg in 9 and Fish oil dose > 500 mg in 26	46 pregnant women in 1^st^ and 3^rd^ trimester	Prenatal fish oil or fish oil supplementation in the first trimester reduced asthma risk among offspring at age 3 y	(94)
n-3 PUFA-rich fish oil	Fish oil capsules (900 mg of LCPUFA) vegetable oil control group	706 children with familial risk of allergy	No difference between fish oil and controls in allergic symptoms or sensitization at 1, 3 and 6 y	(95)
n-3 PUFA-rich fish oil	Fish oil capsules (3.2 g EPA + 2.2 g DHA)/d and placebo	10 athletes with exercise-induced bronchoconstriction (EIB) and 10 athletes without EIB	FEV1 decreased were inhibited (3 ± 2% vs 14.5 ± 5%) at 15 minutes postexercise,TNF-α and IL-1β decreased	(96)
n-3 PUFA-rich fish oil	Fish oil capsules (3.2 g EPA + 2.2 g DHA)/d and placebo	7 male and 15 female atopic and nonsmoking asthma patients (aged 18 - 42 y)	After 2 to 7 h of dietary supplementation with Max-EPA, the late asthmatic response was significantly attenuated (p < 0.05), the EPA content in neutrophil were increased to 10-fold, and neutrophil chemotactic responses were depressed by approximately 50%, 47% inhibition of leukotriene B generation	(97)

**Table 3:** Effects of n-3 PUFAs on inflammation in animal and cellular ‘asthma’ models.

Models	Administration	Effects	Models	Reference
OVA-induced asthma in BALB/c mice and control groups	LCPUFAs (1000 mg/kg/d; 50% EPA and 50% DHA) by gavage from days 21 - 28	Airway response and BALF eosinophils were decreased by LCPUFAs (p < 0.05), IL-5, IL-4, IL-13 levels and remodeling were also decreased (p < 0.05)	OVA-induced asthma in BALB/c mice and control groups	(98)
HDM-induced chronic asthma model in C57BL/6 mice and controls	LCPUFA:1000 mg/kg EPA + 229.6 mg/kg DHA + 246.0 mg/kg GLA + 200.9 mg/kg SDA/day compared to1000 mg/kg/day EPA from days 11 - 35	In asthmatic mouse lung and blood cells, AA and DHA were increased (p < 0.001 and p < 0.01) while DGLA was decreased. (p < 0.05) in lung cells. Combination n-3 and n-6 LCPUFAs decreased AA and increased EPA, DPA (all p < 0.001), and DHA (p < 0.01) and reversed the lack of DGLA (p < 0.05)	HDM-induced chronic asthma model in C57BL/6 mice and controls	(69)
HDM-induced asthma model in C57BL/6 mice and controls		LCPUFA combination reduced AHR, decreased the relative amount of eosinophils, reduced IL-5, IL-4, IL-13, IL-6, IL-10 and IFN-γ, IL-6, and increased the release of EPA derived E-series resolvins (RvEs), and DPA-derived SPMs and D-series resolvins (RvDs) in BALF	HDM-induced asthma model in C57BL/6 mice and controls	(99)
PM2.5-induced lung injury in male C57BL/6N and control mice	ω-3 PUFAs-enriched diet (EPA/DHA = 3:2) 21 g/kg for 6 weeks, with/without intratracheal PM2.5	n-3 fatty acid group showed reduced alveolar septal thickness and inflammatory cells, with decreased levels of TNF-α, IL-1β, IL-6, and IL-17 (p < 0.05 - 0.01) in serum and BALF	PM2.5-induced lung injury in male C57BL/6N and control mice	(100)
HDM-induced asthma model in female adult mice	OmeGo (enzymatically liberated salmon oil; 20 and 60 μg, vehicle and positive control (apolipoprotein)	vehicle and positive total cell and eosinophil countsin BALF (p < 0.01) and eosinophils in spleen (p < 0.001)	HDM-induced asthma model in female adult mice	(101)
Intraperitoneal eosinophilia polymyxin B model in guinea pig	OmeGo (30 mg/kg, 300 mg/kg), sea cod (cod liver oil/omega 3) (30 mg/kg, 300 mg/kg), also fevipiprant 5 and 20 mg/kg, and Linoleic acid (LA) 300 mg/kg	300 mg/kg OmeGo attenuated eosinophil chemotaxis (50.7%, p < 0.002) and chemokinesis (55.7% p < 0.005) to leukotriene B4 compared to LA control	Intraperitoneal eosinophilia polymyxin B model in guinea pig	
LPS-induced acute lung inflammation in male Wistar rats	O3FFA:31.6% EPA, 31.6% DHA, and 15.4% DPA.EE: fish oil concentrate with 22% DHA and 33% EPA and saline and LPS controls	O3FFA and O3EE reduced LPS induced alveolar histiocytosis and decreased BALF IL-6, TNF-α, TGF-β, and IL-10 (p < 0.05)	LPS-induced acute lung inflammation in male Wistar rats	(102)
Human DC2 - T-cell model and LPS matured DCs as a control	100 µM of LA, ALA, AA, DHA, or EPA	DHA (p < 0.005) and EPA (p < 0.0001), LA and AA decreased IL-12/IL-23 and IL-23production by DC2s and lowered IL-13, IFN-γ and IL10 production by DC2-induced effector T-cells	Human DC2 - T-cell model and LPS matured DCs as a control	(103)

OVA ovalbumin, HDM, house dust mite; BALF, bronchoalveolar lavage fluid; DGLA, dihomo-linoleic acid; PM2.5, particulate matter 2.5; AHR, airway responsiveness (to metacholine); LPS, lipopolysaccharide; DC2, dendritic cells; SPM, specialized pro-resolving mediators.

There is evidence suggesting that n-3 PUFAs and marine oils have protective effects against asthma and allergies, as demonstrated in both animal studies and clinical trials (104). As summarized in **Table 1**, n-3 PUFAs were supplemented in a Swedish cohort of children, a French longitudinal study of pregnant women and a small study in children with asthma. The key constituents, particularly DHA and EPA, were often reported in combination, in clinical trials or studies. Generally, n-3 PUFAs were beneficial in improving asthma-induced pathologic changes (85, 86), in reducing levels of inflammatory cytokines (86), and in the reduction in usage of asthma medications (90). In addition, prenatal n-3 PUFAs played a role in prevention of asthma risk in offspring (91, 92, 94). However, n-3 PUFA treatment did not lead to positive or significant results in some clinical reports and the effectiveness and mechanisms of action of n-3 PUFAs require further study. As shown by a meta-analysis, fish intake and maternal n-3 PUFA supplement lowered the asthma risk in childhood, but had no significant effect in adult asthma (105), while in a Cochrane review, including 9 clinical trials, no consistent effect of n-3 PUFAs on asthma was demonstrated, apart from one study indicating a reduction of asthma medication (106). A systematic review of 14 studies reported benefit effects of n-3 PUFAs on T2 inflammation (107). A further review of the effects of n-3 PUFAs on asthma pathology, cytokines and asthma exacerbations also reached similar inconsistent conclusions (108).

As shown in **Table 3**, combined use of different n-3 PUFAs was documented in cell and animal experiments, with attention paid to downstream inflammatory products and signaling mechanisms. Broadly similar results in these animal and cellular experiments were seen to those in clinical trials suggesting protective effects of combined-n-3 PUFAs on pathologic changes in asthma, with a reduction in airway responsiveness (99), reduction in remodeling (100) and attenuation of eosinophil chemotaxis and chemokinesis (101) etc. Inflammatory cytokines, important in asthma, were generally decreased by n-3 PUFAs, particularly the Th2-type cytokines IL-5, IL-13 (98, 103) and those produced by Th1/Th17 cells such as TNF-α, IL-1β, IL-6, IL-17, and IL-23 (100).

## 7 The anti-asthma activity of DHA and DHA-derived lipid mediators: resolvins, maresins and protectin

DHA is the most significant fatty acid of the n-3 family, with much evidence suggesting beneficial effects on airway inflammation and in asthma prevention (76). In a clinical investigation of 91 healthy infants, born between 37- and 42-weeks gestation, fed with 0.32, 0.64, or 0.96% DHA or 0.64% arachidonic acid (ARA) as dietary supplements, a lower incidence of wheezing/asthma resulted, despite the mothers having a history of allergies (109). In the Etude Longitudinale Francaise depuis l’Enfance (ELFE) cohort of 8389 formula-fed infants, a high DHA content resulted in a low risk of wheezing and lower respiratory tract infections, with a lower use of asthma medications (90). DHA in human milk may also reduce allergy risk in the offspring (110).

**Figure 5:**
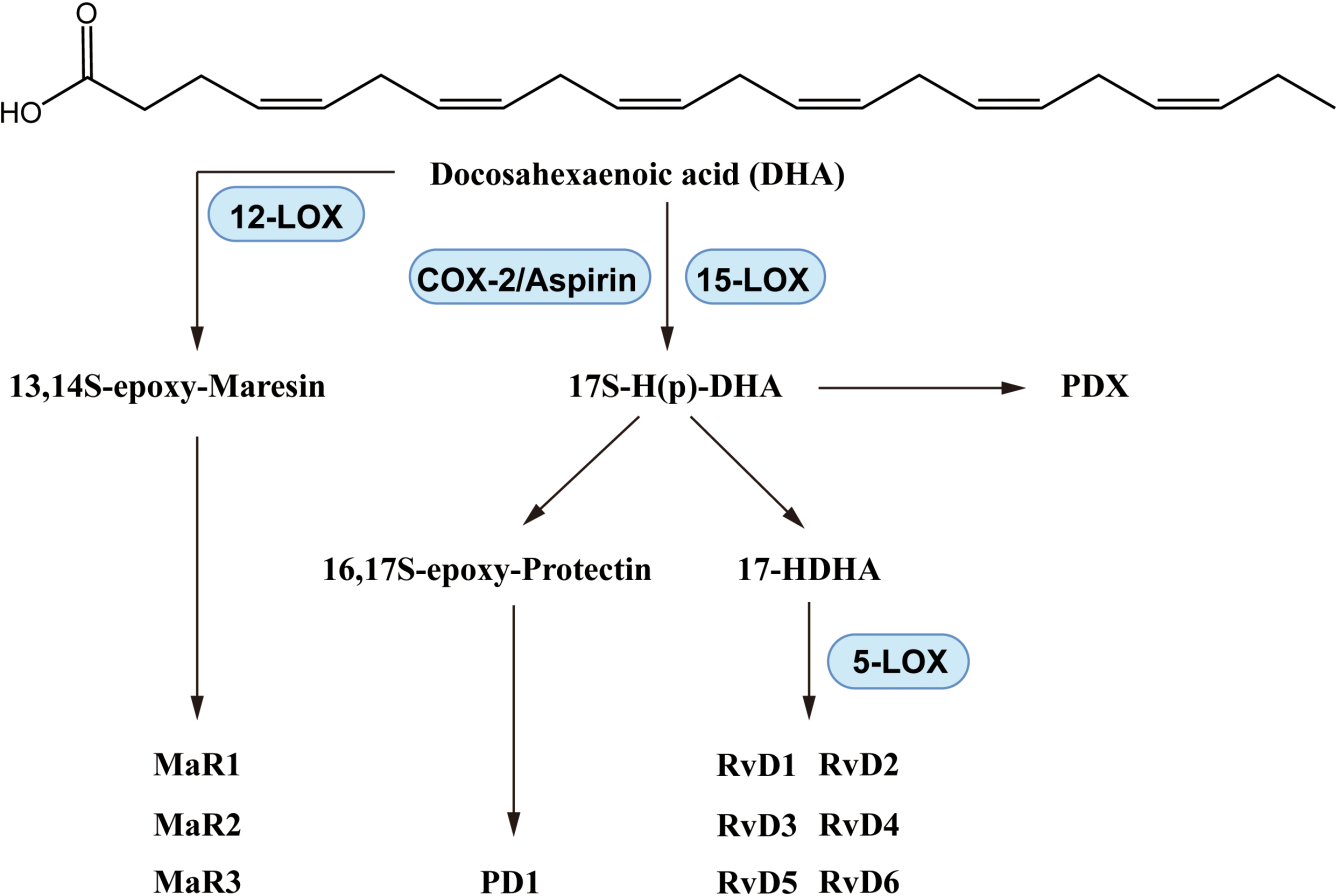
Biosynthesis of DHA-derived lipid mediators.

DHA reduced the pathologic changes of asthma in a mouse model (111), and inhibited prostaglandin F_2α_-induced tracheal smooth muscle contraction (112). In dust-induced lung inflammation in mice, DHA increased levels of Resolvin D (RvD)1, one of the DHA-derived lipid mediators, and inhibited neutrophil and macrophage recruitment (113). In a mouse agricultural dust study, DHA reduced lung neutrophil, macrophage and lymphocyte counts and IL-6 and TNF-α levels in bronchoalveolar lavage fluid (BALF), with increased RvD1 and RvD2 as well as altered macrophage polarization (114). These effects indicated that DHA significantly inhibited macrophage factors induced by lipopolysaccharide (LPS) or SiO_2_, reducing levels of proinflammatory eicosanoids including prostaglandins, leukotrienes, and thromboxane (115). In an agricultural dust-induced BEAS-2B inflammatory cell model DHA reduced levels of IL-6, IL-8 and TNF-α and promoted production of RvD1, amphiregulin and cell injury repair (116). SPMs including resolvins, maresins and protectins are produced from DHA via enzyme mediated biosynthesis as shown in **Figure 5**.

Resolvins, including RvD 1 - 6, were discovered after 2002, synthesized (117–122), and some have been produced on a commercial scale (123). The systemic anti-inflammatory activity of resolvins has been widely reported (124, 125). In asthma, characterized by chronic inflammation, resolvins have also shown beneficial effects. In ovalbumin (OVA)-induced murine asthma, RvD1 reduced BALF eosinophils and lymphocytes, alleviated AHR, and lowered IL-5 and IL-23 levels while enhancing allergen phagocytosis by lung macrophages (126). In children with moderate and severe asthma, RvD1 levels were typically reduced, suggesting that RvD1 might be a potential indicator of asthma severity (127). RvD1 ameliorated LPS-induced lung injury by decreasing neutrophil infiltration and lung TNF-α concentrations (128). RvD1 and RvD2 decreased IL-8 and other factors and promoted IL-10 production, and activated the glycogen synthase kinase-3β anti-inflammatory axis in human monocytes (129). RvD1 and RvD2 inhibited the differentiation of Th1/Th17 cells and promoted production of Tregs through the signature transcription factors T-bet and Rorc (130). An epimer of RvD1, AT-RvD1, has been reported to possess potential anti-asthma activity. AT-RvD1 was found to downregulate TNF-α in the peripheral blood mononuclear cells (PBMCs) from both severe asthma patients and healthy individuals (131). In addition to RvD1 and RvD2, the other RvDs also showed anti-inflammatory activity. RvD3 protected against epithelial lung injury (132) and RvD4 promoted neutrophil apoptosis and neutrophil, monocyte and macrophage phagocytosis (133). RvD5 down-regulated levels of IL-6 and the C-C motif chemokine ligand (CCL)5 in LPS-stimulated THP-1 cells (134). Furthermore, the D-series Resolvins D1-5 activated Phospholipase D, a potential target in phagocytes (135).

Research into mechanisms suggested that the proresolving actions of RvD1 on macrophages, neutrophils and leukocytes were associated with two G protein-coupled receptors (GPR) the formyl peptide receptor 2 (ALX/FPR2) and GPR 32 (136, 137), as with regulation of macrophage polarization into the anti-inflammatory type-M2 type (138, 139). ALX/FPR2 receptors were identified in T cells, macrophages and neutrophils (140, 141), and through the Gi/O family transduction mechanisms, ALX/FPR2 regulated Ca2+ flux by a CD38- dependent cyclic ADP-ribose (142), and influenced the expression of nuclear factor kappa-B (NF-κB) (140). RvD1 and RvD2 inhibited neutrophil apoptosis and promotion of macrophage phagocytosis, and these effects were reversed by GPR32 and ALX/FPR2 antibodies in a mouse LPS model of lung inflammation (143).Furthermore, an ALX/FPR2 inhibitor prevented the RvD1-reduction of TNF-α by preventing the RvD1 stimulation of type-M2 macrophages (144). Additional supportive evidence from a clinical study in severe pediatric asthma reported reduction of lipoxin A4 levels and FPR2/ALX expression (133). AT-RvD promoted phagocytosis of apoptotic neutrophils and downregulated NF-kB; anti-inflammatory effects also mediated by ALX/FRP2 receptors (145).

Maresins exhibit significant anti-inflammatory effects in lung disease. In an OVA-induced asthma model, maresin (MaR)1 alleviated inflammatory cell infiltration, reducing neutrophil and eosinophil counts, and decreasing T2-cytokines by NF-κB inhibition (146). MaR1 reduced levels of IL-5 and IL-13 in lung and ILC2 cells in OVA-induced allergic BALB/c mice. MaR1 lowered IL-6, TNF-α and the production of Tregs in an acute lung injury model (147). In pancreatitis-related lung injury, MaR1 reduced levels of IL-1β, IL-6 and TNF-α and increased IL-10 level in lung tissues (148). The anti-inflammatory activity of MaR1 was associated with the receptor retinoic acid-related orphan receptor α (RORα) and human leucine-rich repeat containing G protein-coupled receptor 6 (LGR6) (149, 150). The effects of MaR2 on Tregs and ILC2 cells were related to LGR6; LGR6-knockout mice showed IL-13 increasing and MaR1 inhibiting effects (151). In human and mouse phagocytes, MaR1 increased phagocytosis which was significantly enhanced by LGR6 overexpression (152). MaR2 decreased the chemokines CCL2, CCL3, CCL17 and other factors in LPS-injured mice (153), and conjugates of MaR1 and MaR3 reduced lung injury (154) and AHR (155).

There is less published research on protectins compared to that on resolvins and maresins, but existing studies have suggested a relationship with asthma and inflammation. Protectin D1 (PD1) administration improved AHR and mucus texture, decreased eosinophil and T-lymphocyte counts, and attenuated lung inflammation in murine asthma (156). An etiological study in infants (157) reported that particulate air pollutants increased asthma susceptibility and decreased PD1 levels. PD1 synthesis was inhibited in eosinophils of patients with severe asthma (158). PD1 downregulated IFN-γ and TNF-α in patients with severe asthma (159), and PD1 alleviated infiltration and extracellular traps of neutrophils with decreased IL-6 and TNF-α in LPS-induced acute lung injury (160). Serhan’s group reported that PD1 promoted leukocyte ingestion and macrophage phagocytosis, and facilitated phagocyte removal in inflammation resolution (161). The PD1 isomer, protectin DX (PDX), was also reported to have anti-inflammatory activity in lung (162). PDX alleviated the symptoms of lung injury in mice (163), increased alveolar fluid clearance in rats (164), and promoted alveolar epithelial cell proliferation (165). PDX inhibited BALF macrophage and neutrophil recruitment in a mouse lung injury model via the TNF-α signaling pathway (166). Protectin conjugates in tissue regeneration (PCTR1) played a protective role in acute LPS lung injury in mice, reduced IL-1β, IL-6 and TNF-α (167). In general, the anti-asthma and anti-inflammatory activity of DHA have been reported in research, with the DHA-derived lipid mediators, including resolvins, maresins and protectins potentially showing beneficial effects in both Th2 and Th1/Th17 immune mechanisms.

## 8 EPA and resolvin Es in asthma

EPA, a key component of n-3 fatty acids, has been studied extensively in asthma and inflammation research. In a double-blind, randomized clinical trial of 35 mild to moderate atopic asthmatics, a medical food emulsion containing EPA and gamma-linolenic acid (GLA) was reported to show improved asthma status in 19% patients with a 23% reduction in rescue medication use (168). In an uncontrolled second study on 65 patients, there was a significant improvement in quality of life questionnaires in asthma patients (p < 0.001). EPA may have beneficial effects on mesenchymal stromal cells in asthma, with reduction in levels of IL-4 and IL-13 and increase in the anti-inflammatory mediator IL-10 (169). The EPA derivative, monoacylglyceride (MAG)-EPA, may reduce bronchial hyperresponsiveness and Ca^2+^ hypersensitivity of bronchial smooth muscle in asthmatic guinea-pigs with reduced eosinophils and lymphocytes and lower transcript counts of eotaxin and related factors (170). Following EPA supplementation, EPA and DPA showed an increase in mice (69).

**Figure 6:**
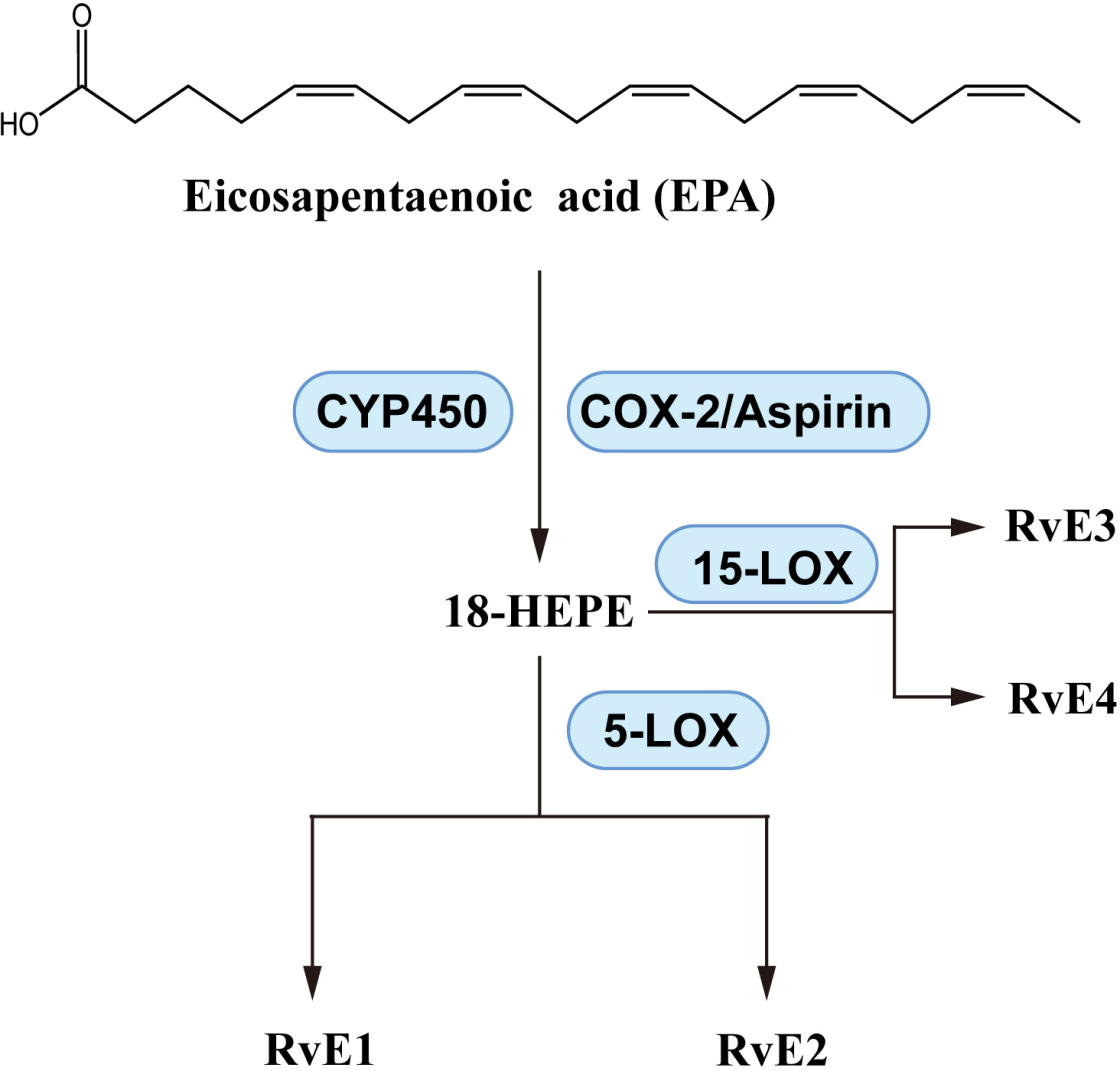
E-series resolvins; biosynthesis pathways.

As shown in **Figure 6**, EPA produces pro-resolvin mediators called E-series resolvins, which consist of RvE1, RvE2, etc. A variety of enzymes play catalytic roles in the production of resolvins, including aspirin-induced acetylated cyclooxygenase-2 (COX-2), cytochrome P450, 5-lipoxygenase (LOX) and 15-LOX (171). EPA is a substrate for E-series resolvins, and supplementation with EPA upregulates the levels of RvEs (99, 172). The laboratory of CN Serhan has been instrumental in elucidating the structure and biosynthesis of RvE1 (173), RvE2 (174), RvE3 (175), RvE4 (176). With reports of anti-inflammatory activity of RvEs (161, 177), there is evidence of beneficial effects of RvEs on asthma and inflammation. In OVA-induced BALB/c mice, RvE1 reduced IL-6, IL-17, IL-23 and improved AHR (178). Even at a dose of 200 ng/day, RvE1 reduced IL-17A and related factors, effectively reduced the eosinophil, macrophage and lymphocyte counts (179). These effects suggest that RvE1 inhibits Th1/Th17 cytokine imbalance of. Targeted research on the effect of RvE1 on Th17 differentiation further elaborated the mechanism, RvE1 suppressed the activation of DCs and T cell, inhibited IL-17 expression with reduction in the levels of IL-17A, IL-21, IL-2 and IL-6 (180).

In asthmatic FVB mice, RvE1 has been reported to decrease IL-13 and immunoglobulin E (IgE) and improve AHR (181). Another study showed similar effects on Th2-type cytokines, decreased IgE, eosinophils and lymphocytes, and related factors in lung and BALF (182). A more comprehensive examination of cytokine levels demonstrated effects of RvE1 on IL-4, IL-5, IL-1β, IL-6, IL-9, IL-13, IL-17, granulocyte macrophage colony-stimulating factor, IFN-γ and CCL family members CCL4, CCL5 and CCL11, restoring BALF cytokine levels to near baseline levels 24 - 36 h after RvE1 administration and also induced Th2 cell differentiation (183). Although relatively little research has been reported, a number of other RvEs have also demonstrated anti-asthma or anti-inflammatory activity. In asthma-susceptible neonatal BALB/c mice, RvE2 reduced eosinophil counts and IL-4, IL-5 and IL-13 levels, suggesting that RvE2 may prevent asthma risk (184). In house dust mite (HDM)-induced allergic mice, RvE3 reduced eosinophils, decreased IL-23 and IL-17 levels in BALF, and downregulated ribonucleic acid (RNA) expression in lung and peri-bronchial lymph nodes (185). In addition, anti-inflammatory activities of RvE3 and RvE4 have been reported in cell experiments (186, 187).

## 9 DPA and DPA-derived resolvins_n-3_ DPA, protectin_n-3_ DPA and Maresinsn-3 DPA

As shown in **Tables 1**, **2**, the combination of DPA with other fatty acids has been used in clinical and animal studies related to asthma. There are very few studies on the use of DPA alone in the treatment of asthma, but there is some literature on the anti-inflammatory effects of DPA and its derivatives. In a model of colitis, DPA inhibited the RNA expression of TNF-α, IL-1β and IL-6 and increased the amount of IL-10 (188). Increased levels of DPA induced by n-3 fatty acids improved TNF-α related apoptosis-inducing ligand and reduced allergic symptoms in infantile mice (189). MAG-DPA, a glycerol esterification product of DPA, downregulated mRNA expression of the TNF-α/NF-κB and COX-2 pathways and controlled the Ca^2+^ sensitivity and airway overactivity in a guinea pig AHR model (190). In experimental pulmonary hypertension, MAG-DPA showed similar anti-inflammatory activity and downregulated NF-κB expression (191). In addition, DPA derivatives were found to decrease TNF-α activity (192, 193).

**Figure 7:**
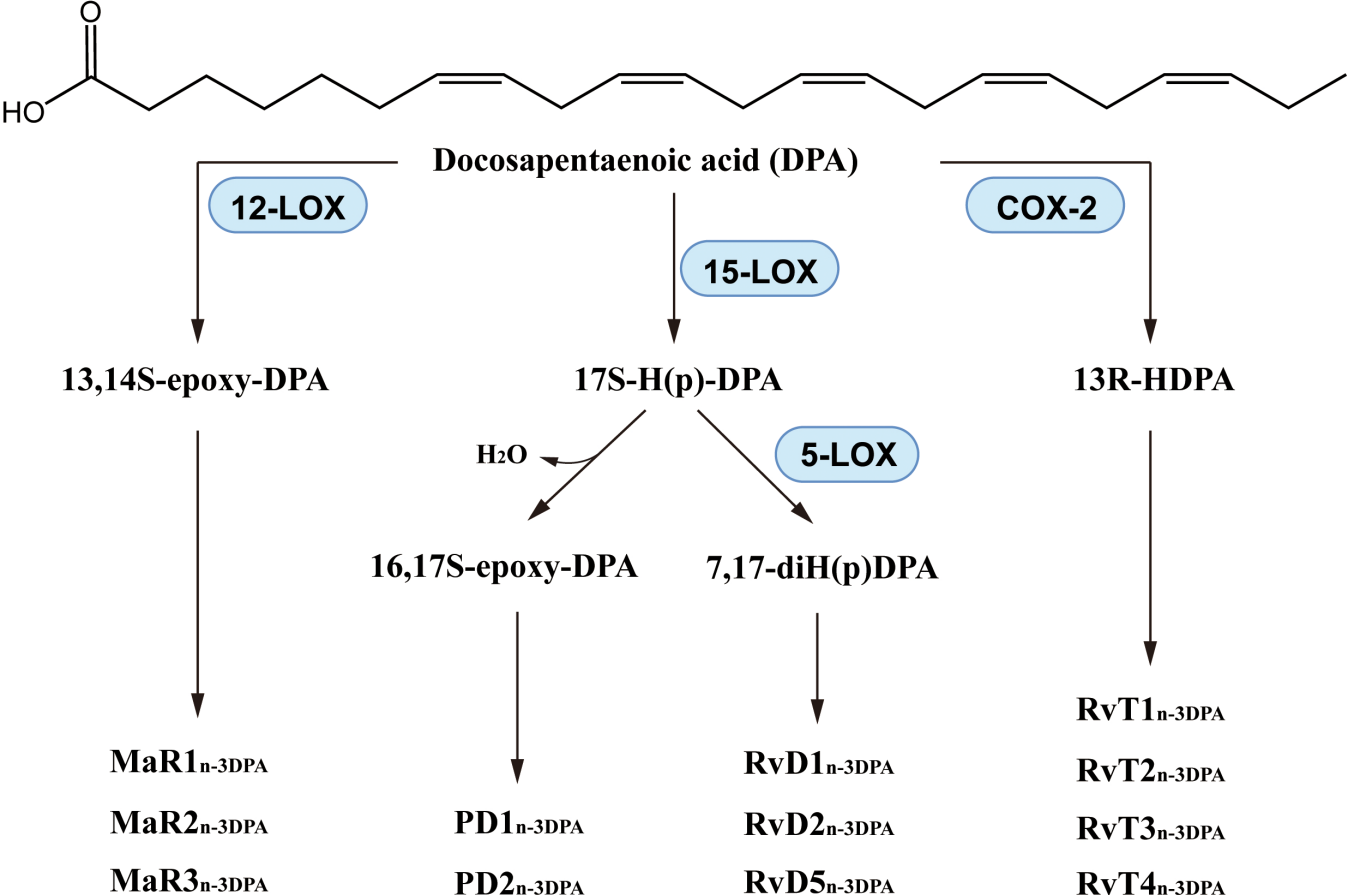
Biosynthesis of DPA-derived lipid mediators.

Through reactions catalyzed by 5-LOX, 15-LOX or other enzymes (194), DPA produces lipid mediators including resolvins_n-3_ DPA, protectins_n-3_ DPA and Maresins_n-3_ DPA (**Figure 7**). It is also worth noting that the production processes of these DPA-derived SPMs is somewhat similar to that of DHA-derived SPMs. In recent years, the synthesis pathway of DPA-derived SPMs has been described (195–200) and the anti-inflammatory activity of these lipid mediators has demonstrated. RvD1_n-3_ DPA decreased neutrophil numbers (195) and reduced NF-κB expression (201). The neutrophil activation marker CD11b was downregulated when plasma RvD1_n-3_ DPA was increased (202). RvD5_n-3_ DPA increased the amount of IL-10 and IL-10R and enhanced phagocytosis of neutrophils and macrophages in murine inflammatory arthritis by a mechanism that may be related to the receptor GPR101 (197, 203, 204). PD1_n-3_ DPA reduced the number of neutrophils and promoted phagocytosis and excretion by macrophages in mice with peritonitis (199). PD1_n-3_ DPA and PD2_n-3_ DPA regulated human monocyte differentiation and macrophage phenotype, and also stimulated phagocytosis in phagocytes, as in mice (205). In addition, PD1_n-3_ DPA and its analogs were protective against neuroinflammation (206) and neuropathic pain (207). 13-series resolvins (also called RvTs) had potent anti-inflammatory effect, that was substantially produced in the initiation phase of inflammation, down-regulating expression of caspase-1 and IL-1β of apoptotic neutrophils and macrophage exudation, RvTs inhibited neutrophil infiltration and improved macrophage uptake of neutrophil extracellular traps, in which the cAMP-PKA-AMPK pathway may be involved (208, 209). RvTs also have a likely treatment role in inflammatory arthritis, and the anti-inflammatory effects of therapeutic agents such as atorvastatin and pravastatin are markedly impaired when the RvT biosynthesis initiating enzyme, COX-2, is inhibited (210).

## 10 Conclusions

**Figure 8:**
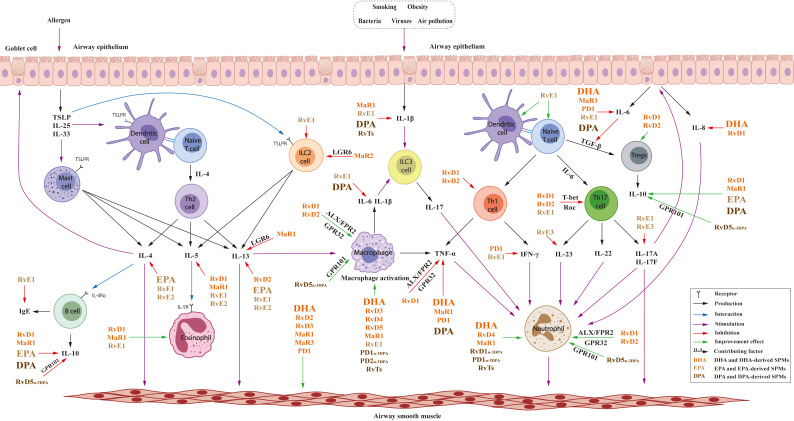
Effects of n-3PUFAs and their lipid mediators in T2 and Th1/Th17 immune.

As shown in **Tables 1**–**3**, the clinical trials and animal experiments indicated the anti-asthma and anti-inflammatory effects of n-3 PUFAs. The combination of n-3 PUFAs and n-3 PUFA-rich diets improved asthma-induced pathologic changes, lowered asthma risk and the use of asthma medication. As summarized in **Figure 8**, DHA, EPA and DPA regulated immune cells including macrophage and neutrophils with effects on the Th2-type cytokines IL-4, IL-13 and cytokines produced by Th1/Th17 including TNF-α, IL-1β, IL-6, IL-8, IL-10 etc. However, there are some different opinions regarding the effects of n-3 PUFAs because of the inconsistent results of some clinical studies. In addition, although n-3 PUFA supplements in pregnancy and early childhood have generally decreased asthma risk, their effects in adults were less obvious. These results suggest the importance of life stages for n-3 PUFA supplementation, and further studies are required to elucidate the mechanisms of action and potential role of n-3 PUFAs in anti-asthma effects.

Further research on n-3 PUFA-derived lipid mediators may offer more insight into their anti-asthma effects. DHA-generated resolvins, maresins and protectins demonstrate similar, but more comprehensive, anti-inflammatory activity compared to DHA, with regulation of IFN-γ, TGF-β and differentiation of Th1 and Th17 cells. The G protein-coupled receptors ALX/FPR2 and GPR32 play important roles in the mechanism of action of RvDs, since the antibody to, and the inhibitor of, these receptors suppressed the anti-inflammatory effects of RvDs and DHA. The anti-inflammatory targets of EPA and RvEs, with effects on IL-4, IL-5, IL-13, are similar but there are some differences. RvEs exert effects on the Th1/Th17 cytokines TNF-α, IL-23, IL-17, and EPA regulate the level of IL-10. Fewer studies on DPA-derived SPMs were reported in the regulation of macrophages and neutrophils. However, the similarities between effects of n-3 PUFAs and their lipid mediators indicate that the lipid mediators may be the active substances, and their inflammation resolution activity may lead to their application in asthma therapy and prevention. In general, supplementation with n-3 PUFAs has been shown to be beneficial as adjunctive therapy for asthma although further study is needed, and SPMs are promising, potential adents for the treatment of asthma.
